# Changes in time-segment specific physical activity between ages 10 and 14 years: A longitudinal observational study

**DOI:** 10.1016/j.jsams.2014.10.003

**Published:** 2016-01

**Authors:** Hannah L. Brooke, Andrew J. Atkin, Kirsten Corder, Ulf Ekelund, Esther M.F. van Sluijs

**Affiliations:** a MRC Epidemiology Unit and UKCRC Centre for Diet and Activity Research (CEDAR), University of Cambridge School of Clinical Medicine, Cambridge, UK; b Department of Sports Medicine, Norwegian School of Sports Sciences, Oslo, Norway

**Keywords:** Child, Adolescents, Accelerometry, Longitudinal studies, Time-segments, Patterns

## Abstract

**Objectives:**

Describe (1) time-segment specific changes in physical activity (PA) into adolescence, (2) differences in change in PA *between* specific time-segments (weekdays–weekends, in-school–out-of-school, out-of-school–weekends, lesson-time–lunch-time), and (3) associations of change in time-segment specific with overall PA.

**Design:**

Longitudinal observational study (4-year follow-up).

**Methods:**

Children from the SPEEDY study (*n* = 769, 42% boys) had PA measured by accelerometer for at least three days at ages 10.2 ± 0.3, 11.2 ± 0.3 and 14.3 ± 0.3 years. Changes in moderate-to-vigorous PA (ΔMVPA, minutes ≥2000 counts/minute [cpm]) and total PA (ΔTPA, average cpm) during weekdays, weekends, in-school, out-of-school, lesson-times and lunch-times, were tested using three level (age, individual, school) mixed-effects linear regression. Differences in ΔMVPA/ΔTPA between time-segments were tested using time-segment × age interaction terms. Associations of four-year time-segment specific ΔMVPA/ΔTPA with four-year overall ΔMVPA/ΔTPA were tested using two level (time-segment specific ΔMVPA/ΔTPA, school) mixed-effects linear regression.

**Results:**

MVPA and TPA declined in all time-segments, except lesson-time MVPA. Annual ΔMVPA and, for boys only, ΔTPA was greater on weekends than weekdays (beta ± SE for interaction term: boys, −3.53 ± 0.83 min, −29.64 ± 7.64 cpm; girls, −2.20 ± 0.64 min) and out-of-school (boys, −4.36 ± 0.79 min, −19.36 ± 8.46 cpm; girls, −2.44 ± 0.63 min). ΔMVPA and ΔTPA during lunch-time was greater than during lesson-time (boys, −0.96 ± 0.20 min, −36.43 ± 6.55 cpm; girls, −0.90 ± 0.13 min, −38.72 ± 4.40 cpm). ΔTPA was greater out-of-school than in-school (boys, −19.89 ± 6.71 cpm; girls, −18.46 ± 6.51 cpm). For all time-segments, four-year ΔMVPA/ΔTPA was positively associated with four-year overall ΔMVPA/ΔTPA (all *p* < 0.042), except for girl's in-school and lunch-time TPA.

**Conclusions:**

Interventions focused on PA maintenance could target all time-segments, but weekends and out-of-school may be particularly advantageous due to the relatively large declines observed.

## 1 Introduction

Physical activity (PA) is important for young people's health,^1^ however, activity levels are often low and are reported to decline from childhood to adolescence.^2^, ^3^ Interventions focused on maintaining PA in youth may help confront the public health challenges associated with insufficient activity. Interventions to date have had relatively small effects on children's PA^4^; further research may help optimise future interventions.^5^

Evidence of the social, psychological and environmental factors which influence PA helps inform intervention design.^6^ Some studies suggest that these factors vary within- and between-days.^7^, ^8^, ^9^ Investigating PA accumulated over specific times of the day or week is therefore vital. A recent systematic review of cross-sectional data found that children were more active during some time-periods than others.^10^ However, longitudinal work is necessary to identify during which periods PA declines, and whether there are greater declines in some time-periods than others. In interventions aimed at PA maintenance there may be greater possibility to influence behaviour in the time-segments with the greatest decline.

Significant declines in PA have been shown on weekdays and weekend days,^3^, ^11^ but results have not been consistent.^12^, ^13^ Significant declines have also been reported after-school,^11^ in-school, before school, at recess and after-school,^12^ and during recess and lunch-time.^14^ However, limited replication of analyses and inconsistencies in time-segment definitions hinders data synthesis. We have previously shown a greater decline in PA on weekend days than weekdays^15^; no other study, to our knowledge, has tested differences *between* time-segments in the magnitude of the decline in PA. Examining differences in time-segment specific change in PA, beyond a simple weekday/weekend comparison, will provide a more complete characterisation of changes in children's activity. It will also help to inform intervention design and ensure that limited resources are utilised effectively. For example, the greatest declines in children's activity may be after-school and on weekends^16^; comparing the decline in activity in these time-segments may indicate which of these periods has most potential as an intervention target. Moreover, if in-school activity is found to decline, determining whether there is a greater decline during lesson-time or lunch-time will aid decision-making during intervention design. In addition, while associations of time-segment specific PA with overall PA have been explored in cross-sectional data^17^ investigating these associations in longitudinal data, and for a wide selection of time-segments, will further support efficient intervention design.

We aimed to describe (1) time-segment specific changes in PA from childhood to adolescence, (2) differences in change in PA between time-segments (weekdays vs. weekends, in-school vs. out-of-school, out-of-school vs. weekends, and lesson-time vs. lunch-time), and (3) associations of changes in time-segment specific PA with changes in overall PA.

## 2 Methods

The Sport, Physical activity and Eating behaviour: Environmental Determinants in Young people (SPEEDY) study is a longitudinal cohort study, which has been described previously.^18^ Full ethical approval was granted by the University of East Anglia local research ethics committee.

At baseline, primary schools in Norfolk, Eastern England, were purposively sampled to achieve heterogeneity in urban/rural location. School with ≤12 Year-5 pupils were excluded for logistical reasons. Within locational strata schools were randomly selected. Of 227 eligible schools, 157 (69%) were invited to the study to achieve the proposed target of *n* = 2000 participants. Ninety-two schools agreed to participate and all children in Year-5 were invited to take part. Schools were broadly representative of all eligible schools, but included fewer schools in urban areas and independent schools due to the sampling frame.^18^ At all schools, class presentations were held to introduce the study and reminder letters were issued. Overall, 2064 pupils returned written informed parental consent and were willing to participate (57% of eligible sample). Trained research assistants visited schools from April to July 2007 and conducted anthropometric measurements, supervised the completion of questionnaires and fitted each child with an accelerometer. Children took a questionnaire home for their parent or guardian to complete; this was to be returned to their school, along with the accelerometer, eight days later.

One-year follow-up data collection took place from April to July 2008. Information packs and consent forms were sent to the home address of children who participated at baseline (up to 3 times). The 1019 children (49% of baseline sample) who provided written parental consent were sent an accelerometer, which they were asked to wear for seven days and return by post using an addressed, pre-paid envelope. They were then entered into a prize draw to win shopping vouchers worth £25.

Four-year follow-up data collection was conducted from April to July 2011. Participants with an active postal address and who had not withdrawn from the study were contacted via their home address up to 3 times (*n* = 1964, 95% of baseline sample). Overall, 415 children provided written parental consent to participate (20% of baseline sample). Trained research assistants visited these children at school to conduct anthropometric measurements and fit accelerometers; these were returned to school after one week. Participants were offered £10 worth of shopping vouchers upon monitor return.

ActiGraph accelerometers (GT1M, Actigraph LCC, Pensacola, US) were used to assess PA. Children were asked to wear the accelerometer on an elastic waistband on the right hip, while awake, for seven days, only removing it to bath, shower or swim. At all measurement waves, participants and their parents were given a written instruction sheet and reminder letters were issued to encourage monitor return. Data were stored at 5-s epochs and processed using MAHUffe software (http://www.mrc-epid.cam.ac.uk). Our processes were similar to other studies in young people^19^, ^20^, ^21^ and the same at all measurement waves. Data from the day the accelerometer was fitted, periods with >10 min of sustained zero counts (‘non wear time’) and counts before 6 am and after 11 pm each day were removed from all files.

We defined moderate-to-vigorous intensity PA (MVPA) as time (minutes) spent at ≥2000 counts per minute (cpm) in each time-segment. This is approximately equivalent to walking at 4 km/h in children^22^ and has been used in previous studies of children.^23^ Average accelerometer cpm was used as a marker of total PA (TPA); it was calculated as total counts in each time-segment divided by total valid monitoring time in each time-segment.

Time-segment specific PA was derived from accelerometer data summarised for each hour. Time-segments were defined as weekdays (Monday–Friday; 0600–2300), weekend days (Saturday–Sunday; 0600–2300), in-school (Monday–Friday; 0900–1500), out-of-school (Monday–Friday; 0600–0900 and 1500–2300), lesson-time (Monday–Friday; 0900–1200 and 1400–1500) and lunch-time (Monday–Friday; 1200–1400) at all measurement waves. Four-year change in overall PA and time-segment specific PA was calculated as follow-up (2011) minus baseline (2007).

Date of birth was self-reported and age was calculated from the measurement date for each wave. At baseline and four-year follow-up height was measured to the nearest 0.1 cm (Leicester height measure, Chasmors Ltd., Leicester, UK) and weight was measured to the nearest 0.1 kg using a non-segmental bio-impedance scale (Tanita, type TBF-300A, Tokyo, Japan) with children dressed in light clothing without shoes. Body mass index (BMI) (kg/m^2^) was calculated and sex- and age-standardised BMI was derived. Obesity status was dichotomised (non-overweight vs. overweight/obese) based on sex- and age-dependent cut points.^24^

At baseline a parent/guardian self-reported their highest educational qualification; this was used as a proxy measure of socioeconomic status. A categorical variable (parent/guardian education level) was created with 3 groups: ‘GCSE or lower’ (i.e. no educational qualification, school leaving certificate, General Certificate of Secondary Education; GCSE, or equivalent), ‘A-level or lower vocational training’ and ‘University or higher vocational training’.

Children with ≥500 min of accelerometer data on at least three days, including a weekend day, at baseline and at least one follow-up data collection, were included in the main analytical sample. Three days of monitoring has given a reliability coefficient of 0.70 in similar aged children,^21^, ^25^ weekend data were required to examine the weekend time-segment. Overall, 769 children were included in the main analytical sample, this comprised of *n* = 222 with data at all measurement waves, *n* = 482 with data at baseline and one-year follow-up only, and *n* = 65 with data at baseline and four-year follow-up only. Children with valid data at baseline and four-year follow-up, who also had baseline data for BMI and parent/guardian education level (*n* = 279), were used in analyses of four-year change in PA.

Baseline differences in demographic and anthropometric characteristics between those included and excluded from the main analytical sample and the sample used in the analyses of four-year change in activity, and sex differences between those who were included, were tested using linear regression (continuous outcomes) or multinomial logistic regression (categorical outcomes). Robust standard errors, accounting for the potential clustering of children within the same school, were calculated. Sex differences in time-segment specific PA at each measurement wave were tested using two level mixed effects linear regression models with levels: sex and baseline school.

All outcome variables and their residuals were normally distributed. Models were run separately for MVPA and TPA. Preliminary analyses indicated sex by age interactions so all further analyses were stratified by sex. Mean annual change in PA in each time-segment was tested using three level mixed effects linear regression with levels: age, individual and baseline school. The beta coefficients in these analyses represent change in PA for every one-year increase in age. Simple models and models adjusted for baseline PA and time-segment specific accelerometer wear-time were assessed. Differences in annual change in PA between pairs of time-segments (weekdays vs. weekends, in-school vs. out-of-school, out-of-school vs. weekends, and lesson-time vs. lunch-time) were examined using an age by time-segment (binary dummy variable) interaction term. To test the association of four-year change in time-segment specific PA with four-year change in overall PA, two level mixed effects linear regression models with levels: (1) time-segment specific change in PA and (2) baseline school, were conducted. Simple models and models adjusted for baseline age, baseline age- and sex-standardised BMI, parent/guardian education level, baseline MVPA or TPA depending on the outcome, baseline accelerometer wear-time, and accelerometer wear-time measured at four-year follow-up were evaluated.

## 3 Results

Children included in the main analytical sample (*n* = 769) and the sample used to examine four-year change in activity (*n* = 279) had similar baseline personal, anthropometric, demographic and PA characteristics to those who were not included (*n* = 1295 or *n* = 1785). However, relative to those in the main analytical sample, excluded children were older (*p*-value = 0.009), less likely to have parent/guardians with “University or higher vocational training” (*p*-value = 0.015) and had higher baseline MVPA in school and at lunch-time (*p*-values = 0.04 and 0.01, respectively). Children who were not included in the sample used to examine four-year change in activity were less likely to have parents/guardians with “University or higher vocational training” or ‘A-level or lower vocational training’ (*p*-value = 0.01 and <0.001, respectively).

In the main analytical sample and in the sample used in analyses of four-year change in activity, boys and girls had similar personal, anthropometric and demographic characteristics (Supplementary Table 1). PA was higher in boys than girls in all time-segments at baseline, and in all time-segments at follow-up, except weekends and out-of-school (Supplementary Figure 1).

MVPA and TPA declined with age for boys and girls in all time-segments, except MVPA during lesson-time (Table 1). Declines in PA varied between time-segments, from a 30 ± 10 s (beta ± SE) annual decline for in-school MVPA in girls, to a 6 min ± 41 s annual decline for weekend MVPA in boys. The largest annual declines in TPA were also on weekends, on average 63 ± 7 cpm for boys, and 51 ± 7 cpm for girls.

Boys and girls had a greater annual decline in MVPA on weekend days compared with weekdays, and out-of-school, and during lunch-times compared with lesson-times (Table 2). The annual declines of in-school and out-of-school MVPA did not differ, for boys or girls. TPA declined more for boys at the weekend compared with weekdays, and out-of-school. For boys and girls there was a greater annual decline in TPA out-of-school compared with in-school and at lunch-times compared with lesson-times (Table 2).

There were small positive associations of four-year change in time-segment specific PA with four-year change in overall PA for all time-segments, except for girl's in-school and lunch-time TPA (Table 3).

## 4 Discussion

Our results indicate that between age 10 and 14 years PA declines in all time-segments, except lesson-times. Greater declines were found on weekends, out-of-school and during lunch-times compared with other periods. For all time-segments four-year change in PA was associated with change in overall PA. These results may be used to inform the timing and content of future interventions targeting PA maintenance in young people.

Our results are consistent with studies indicating a marked age-related decline in overall PA^26^, ^27^ and provide in-depth understanding of how and when this decline occurs. The findings are supported by studies reporting significant declines in PA on weekdays and weekend days,^3^, ^11^ after-school,^11^, ^13^ in-school, before school, and during recess,^12^ and lunch-time.^14^ However, comparisons between studies are somewhat limited by heterogeneity in time-segment definitions. Some studies report using 1530 to 2030, or end of school until 1800, as ‘after-school’ but do not report other timings,^11^, ^13^ others based their time-segments on ‘school bell times’,^14^ or state timings which may be contextually appropriate but not relevant to the current study.^12^ Although lesson-time TPA was found to decline, lesson-time MVPA did not change with age. The decline in lesson-time TPA may be due to light intensity activity becoming lighter or being replaced by sedentary behaviour. Lesson-time MVPA was low at baseline so the potential for change was limited. In addition, children are largely required to be seated in class, so accumulating MVPA in lesson-times may be difficult for children at all ages. Despite this, high quality physical education may contribute to children's PA, so lesson-time should remain a potential intervention target. Indeed, variance introduced by participation in physical education could have also impacted on the results for lesson-time MVPA.

PA declined to a greater extent at weekends, out-of-school and at lunch-times, compared with weekdays, in-school and lesson-times. Targeting PA maintenance interventions during these time-segments with the greatest declines may be advantageous. Due to the time-limited nature of lunch-times on school-days, and because change in PA only differed between out-of-school and in-school for TPA, targeting weekends may have the greatest overall impact, particularly as children are less likely to be restricted by other responsibilities, such as homework, at the weekend. Moreover, differences in school timetables between measurement waves may have contributed to the greater decline in activity out-of-school and at lunch-time compared with in-school and lesson-time.

The positive associations of change in time-segment specific PA with change in overall PA indicate that interventions targeting any time-segment could potentially influence overall PA. When four-year change in MVPA was the outcome, the strongest associations were for change in lesson-time and lunch-time MVPA and the weakest was for change in weekend day activity. This was surprising given that the greatest decline in time-segment specific activity was on weekends. Despite this, these results indicate that an intervention enabling children to maintain their lesson-time and lunch-time activity levels may also result in maintenance of overall activity. However, these results should be considered in conjunction with the other findings discussed above. For example, on average lesson-time MVPA did not decrease over time so there may be limited scope to target interventions at lesson-time MVPA. Nonetheless, school-time intervention approaches provide access to population subgroups, such as ethnic minorities and socioeconomic disadvantaged children who may be otherwise hard to reach. Interventions should be developed taking into account such factors, as well as evidence from previous time-segment specific interventions.

Strengths of this study include the longitudinal design, a population-based recruitment strategy and objectively measured PA. However, accelerometers cannot be worn for water-based activities and have limited ability to detect activities with little vertical hip movement. Despite implementing strategies that reportedly improve participant retention, such as offering incentives and sending reminder letters,^28^ there was relatively high drop-out between measurement waves. Other studies have encountered similar challenges, for example the 5-year retention rate in the Children Living in Active Neighbourhoods (CLAN) Study was 34%.^29^ Further research into recruitment and retention strategies for adolescents may be warranted to improve these figures. Children included in the main analyses and analyses of four-year change in PA had largely similar baseline characteristics to those who were excluded. However, excluded individuals had higher baseline MVPA in-school and at lunch-time. The estimated changes in activity level in these time-segments are therefore likely to be conservative, as those who were excluded had greater scope to change than those who were included. The potential for bias introduced by the low retention rate in this study means that the results should be generalised to other populations with caution. Moreover, three valid days of accelerometer data, including at least one weekend day, were required. These data were subsequently split into weekdays and weekend days; as such, time-segment specific analyses do not include three valid days of data for all individuals. The timetable followed by some schools did not completely match with our time-segment definitions. The degree of mismatch differed substantially between schools, but no school had a lunch period from 1200 to 1400. We acknowledge this compromise in precision; however, our definitions enabled standard timings to be applied across all schools, accommodated hourly-level accelerometer data, and ensured misclassification only occurred in one direction. Furthermore, misclassification between time-segments is likely to introduce random-error and reduce effect sizes leading to conservative estimates. In addition, we primarily examined changes in PA within time-segments and so the time-segment definitions, which remained constant for all measurement waves, are likely to have had limited influence on the results. In future research assessing time-segment specific PA it may be beneficial to use data summarised over 5 or 10 min periods rather than for each hour, and to consider using individual school timetables to define time-segments more precisely.

## 5 Conclusion

Interventions targeting PA in any period of the day may impact upon overall activity levels. However, the greatest opportunity to effect change may be at weekends, out-of-school and lunch-time, as the decline in PA during these periods was greater than in others, and there may be fewer restrictions on children's use of time.

## Practical implications

• PA interventions may be enhanced by targeting correlates of activity at specific times of the day or week.
• PA monitoring in youth should consider changes in activity within specific periods of the day and week in addition to overall patterns.
• Weekends, out-of-school and lunch-times may present the greatest opportunity to effect change in PA levels.

## Figures and Tables

**Supplementary Fig. I:**
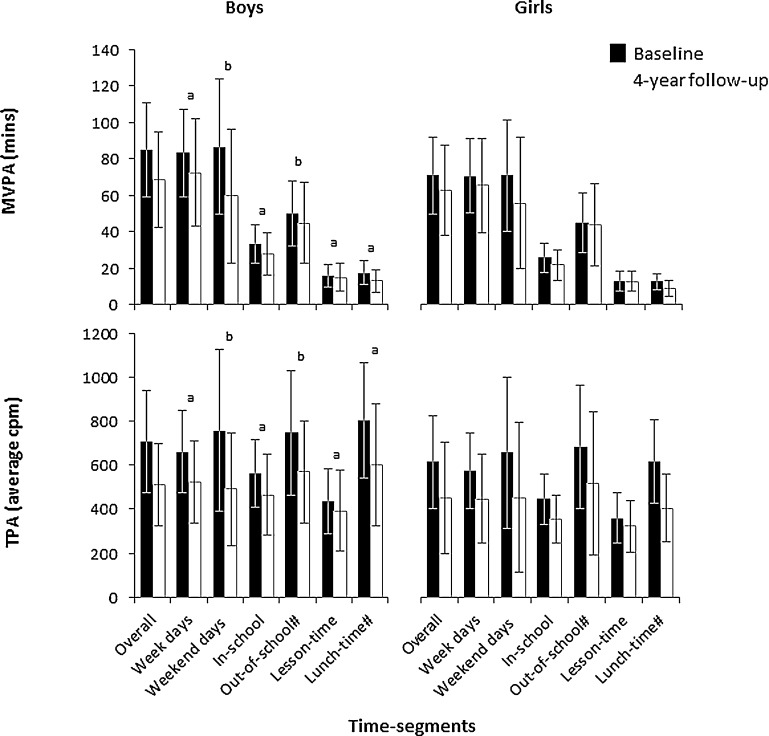
Time-segment specific PA in boys and girls. Data illustrate mean PA in each time-segment, error bars indicate standard deviation. Data are from children with 3 valid days of PA data including a weekend, at baseline (*n* = 769; 446 girls, 323 boys) and four-year follow-up (*n* = 279; 152 girls, 127 boys). Sex differences in time-segment specific PA at each measurement wave were tested using two level mixed effects linear regression models with levels: sex and baseline school. a, sex difference at baseline and at four-year follow-up, *p* < 0.05. b, sex difference at baseline *p* < 0.05 but not at four-year follow-up. #on school days. MVPA, moderate-to-vigorous intensity PA; TPA, total PA; cpm, counts per minute.

**Table 1:** Mean annual change in PA in each time-segment for boys and girls, beta coefficients and 95% confidence intervals.

	Boys (*n* = 323)			Girls (*n* = 446)		
	*β* Coef.	95% CI	*p*-value	*β* Coef.	95% CI	*p*-value
**MVPA (average min/day)**						
Weekdays	−2.44	(−3.33, −1.54)	<0.001	−1.02	(−1.68, −0.36)	0.003
Weekend days	−6.01	(−7.36, −4.65)	<0.001	−3.27	(−4.32, −2.22)	<0.001
In-school	−0.63	(−1.12, −0.15)	0.010	−0.49	(−0.80, −0.17)	0.002
Out-of-school^a^	−1.65	(−2.33, −0.97)	<0.001	−0.77	(−1.32, −0.21)	0.007
Lesson-time	0.00	(−0.33, 0.34)	0.989	0.22	(0.00, 0.43)	0.052
Lunch-time^a^	−0.84	(−1.09, −0.60)	<0.001	−0.70	(−0.86, −0.54)	<0.001
**TPA (average cpm)**						
Weekdays	−32.57	(−39.56, −25.59)	<0.001	−29.13	(−35.91, −22.35)	<0.001
Weekend days	−63.42	(−76.21, −50.63)	<0.001	−50.72	(−63.41, −38.02)	<0.001
In-school	−18.85	(−26.25, −11.44)	<0.001	−23.85	(−28.83, −18.86)	<0.001
Out-of-school^a^	−43.33	(−53.93, −32.74)	<0.001	−39.95	(−51.18, −28.73)	<0.001
Lesson-time	−9.48	(−17.32, −1.65)	0.018	−9.57	(−14.86, −4.28)	<0.001
Lunch-time^a^	−44.71	(−55.31, −34.10)	<0.001	−50.15	(−57.55, −42.75)	<0.001

*β* Coef., beta-coefficient; 95% CI, 95% confidence interval; MVPA, moderate-to-vigorous intensity PA; TPA, total PA; cpm, counts per minute. Associations were tested using three level mixed effects linear regression with levels age, individual and baseline school. Beta coefficients represent change in time-segment specific PA for every one-year increase in age. Children with 3 valid days of PA data including a weekend day at baseline and at least one other time point were included in the models (*n* = 769; this comprised of *n* = 222 with data at all measurement waves, *n* = 482 with data at baseline and one-year follow-up only, and *n* = 65 with data at baseline and four-year follow-up only). Models were adjusted for baseline PA and time-segment specific accelerometer wear-time.

a On school days.

**Table 2:** Comparative annual change in PA between pairs of time-segments (age by time-segment interaction) for boys and girls, beta coefficients and 95% confidence intervals.

	Boys (*n* = 323)			Girls (*n* = 446)		
	*β* Coef.	95% CI	*p*-value	*β* Coef.	95% CI	*p*-value
**MVPA (average min/day)**						
Weekend days (ref: Weekdays)	−3.53	(−5.16, −1.90)	<0.001	−2.20	(−3.46, −0.94)	0.001
Out-of-school^a^ (ref: In-school)	−0.85	(−1.71, 0.01)	0.052	−0.36	(−1.04, 0.32)	0.306
Weekend days (ref: Out-of-school^a^)	−4.36	(−5.90, −2.82)	<0.001	−2.44	(−3.67, −1.21)	<0.001
Lunch-time^a^ (ref: Lesson-time)	−0.96	(−1.36, −0.57)	<0.001	−0.90	(−1.16, −0.65)	<0.001
**TPA (average cpm)**						
Weekend days (ref: Weekdays)	−29.64	(−44.61, −14.67)	<0.001	b		
Out-of-school^a^ (ref: In-school)	−19.89	(−33.04, −6.74)	0.003	−18.46	(−31.21, −5.70)	0.005
Weekend days (ref: Out-of-school^a^)	−19.36	(−35.94, −2.77)	0.022	−11.15	(−28.09, 5.80)	0.197
Lunch-time^a^ (ref: Lesson-time)	−36.43	(−49.27, −23.58)	<0.001	−38.72	(−47.35, −30.08)	<0.001

*β* Coef., beta-coefficient; 95% CI, 95% confidence interval; MVPA, moderate-to-vigorous intensity PA; TPA, total PA; cpm, counts per minute. Associations were tested using three level mixed effects linear regression with levels age, individual and baseline school. Beta coefficients represent the difference in change in PA between time-segments for every one-year increase in age. Children with 3 valid days of PA data including a weekend day at baseline and at least one other time point (*n* = 769) were included in the models. Models were adjusted for baseline PA and time-segment specific accelerometer wear-time.

a On school days.

b Model did not converge.

**Table 3:** Association of four-year change in time-segment specific PA with four-year change in overall PA for boys and girls, beta coefficients and 95% confidence intervals.

	Boys (*n* = 127)			Girls (*n* = 152)		
	*β* Coef.	95% CI	*p*-value	*β* Coef.	95% CI	*p*-value
**MVPA (average min/day)**						
Weekdays	0.61	(0.51, 0.71)	<0.001	0.74	(0.64, 0.83)	<0.001
Weekend days	0.36	(0.28, 0.44)	<0.001	0.43	(0.37, 0.48)	<0.001
In-school	0.77	(0.48, 1.06)	<0.001	0.95	(0.53, 1.36)	<0.001
Out-of-school^a^	0.74	(0.61, 0.88)	<0.001	0.78	(0.66, 0.89)	<0.001
Lesson-time	0.96	(0.54, 1.39)	<0.001	1.44	(0.77, 2.11)	<0.001
Lunch-time^a^	0.94	(0.41, 1.48)	0.001	1.05	(0.34, 1.76)	0.004
**TPA (average cpm)**						
Weekdays	0.44	(0.32, 0.57)	<0.001	0.87	(0.76, 0.99)	<0.001
Weekend days	0.44	(0.38, 0.50)	<0.001	0.61	(0.57, 0.65)	<0.001
In-school	0.36	(0.21, 0.50)	<0.001	0.26	(−0.06, 0.58)	0.114
Out-of-school^a^	0.24	(0.15, 0.33)	<0.001	0.51	(0.44, 0.57)	<0.001
Lesson-time	0.27	(0.12, 0.41)	<0.001	0.35	(0.01, 0.68)	0.041
Lunch-time^a^	0.19	(0.10, 0.28)	<0.001	0.05	(−0.14, 0.24)	0.595

*β* Coef., beta-coefficient; 95% CI, 95% confidence interval; MVPA, moderate-to-vigorous intensity PA; TPA, total PA; cpm, counts per minute. Beta coefficients represent the change in overall PA for every one-minute or one cpm change in time-segments specific PA. Associations were tested using two level mixed effects linear regression with levels time-segment specific change in PA and baseline school. Children with 3 valid days of PA data including a weekend day at baseline and at 4 year follow-up who also had baseline data for BMI and parent/guardian education level were included in change analyses (*n* = 279). Models were adjusted for baseline age, baseline age-and sex-standardised BMI, parent/guardian education level, baseline PA, baseline accelerometer wear-time and accelerometer wear-time at four-year follow-up.

a On school days.

## Appendix A Supplementary data

Table S1 Personal, anthropometric and demographic characteristics of participants, mean (SD) or *n* (%).
