# Supplementary material for: Changes in time-segment specific physical activity between ages 10 and 14 years: A longitudinal observational study
Source: J Sci Med Sport. 2016 Jan;19(1):29–34. doi: 10.1016/j.jsams.2014.10.003 (PMC4678171; doi:10.1016/j.jsams.2014.10.003)
Supplement: Table S1 — Personal, anthropometric and demographic characteristics of participants, mean (SD) or n (%). [file mmc1.docx]

**Supplementary Material**

**Supplementary table 1.** Personal, anthropometric and demographic characteristics of participants, mean (SD) or n (%)

|  | | **Baseline*** | **1-year follow-up*** | **4-year follow-up*** | **Baseline data from children included in 4-year change analyses**** |
| --- | --- | --- | --- | --- | --- |
| **Sex** | |  |  |  |  |
|  | Girls (N) | 446 (58.0) | 410 (58.2) | 154 (53.7) | 152 (54.5) |
|  | Boys (N) | 323 (42.0) | 294 (41.8) | 133 (46.3) | 127 (45.5) |
| **Age (years)** | | 10.2 (0.3) | 11.2 (0.3) | 14.3 (0.3) | 10.2 (0.3) |
| **Height (cm)** | | 140.5 (6.7) |  | 164.4 (7.4)^‡^ | 140.4 (6.4) |
| **Weight (kg)** | | 36.1 (8.3)^‡^ |  | 56.3 (12.2) | 35.7 (8.2) |
| **BMI (kg/m^2^)** | | 18.1 (3.1)^‡^ |  | 20.8 (4.0)^‡^ | 17.9 (3.1) |
| **BMI z-score** | | 0.3 (1.1) |  | 0.3 (1.2) | 0.3 (1.1) |
| **Weight status** | |  |  |  |  |
|  | Non-overweight | 598 (78.2) |  | 232 (81.1) | 224 (80.3) |
|  | Overweight/obese | 167 (21.9)^‡^ |  | 54 (18.9) | 55 (19.7) |
| **Parent/guardian education level** | |  |  |  |  |
|  | GCSE or lower | 277 (37.1) |  |  | 79 (28.3) |
|  | A-level or lower vocational | 295 (39.5) |  |  | 136 (48.8) |
|  | University or higher vocational | 175 (23.4) |  |  | 64 (22.9) |

Data are presented as mean (SD) for continuous variables and n (%) for categorical variables.

*Children with 3 valid days of PA data including a weekend day at baseline and at least one other time point were included in the main analyses (n= 769; 222 children had valid PA data at all three measurement waves, 482 children had valid data at baseline and 1 year follow-up and 65 children had valid data at baseline and four year follow-up).

** Children with 3 valid days of PA data including a weekend day at baseline and 4 year follow-up who also had baseline data for BMI and Parent/guardian education level were included in analyses examining predictors of four-year change in overall activity (n=279).

^‡^Significant difference between boys and girls tested using multilevel linear or multinomial logistic regression according to whether the dependent variable was continuous or categorical.

BMI, body mass index; GCSE, General certificate of secondary education

**Supplementary figure 1**. Time-segment specific PA in boys and girls

Data illustrate mean PA in each time-segment, error bars indicate standard deviation.

Data are from children with 3 valid days of PA data including a weekend, at baseline (n= 769; 446 girls, 323 boys) and four-year follow-up (n=279; 152 girls, 127 boys).

Sex differences in time-segment specific PA at each measurement wave were tested using two level mixed effects linear regression models with levels: sex and baseline school.

a, sex difference at baseline and at four-year follow-up p<0.05.

b, sex difference at baseline p<0.05 but not at four-year follow-up.

^#^ on school days.

MVPA, moderate-to-vigorous intensity PA; TPA, total PA; cpm, counts per minute.
